# Fault Detection and Exclusion Method for a Deeply Integrated BDS/INS System

**DOI:** 10.3390/s20071844

**Published:** 2020-03-26

**Authors:** Junren Sun, Zun Niu, Bocheng Zhu

**Affiliations:** Department of Electronics, Peking University, Beijing 100871, China

**Keywords:** fault detection and exclusion, RAIM, BDS, INS, deep integration, vector tracking loop

## Abstract

The Inertial Navigation System (INS) is often fused with the Global Navigation Satellite System (GNSS) to provide more robust and superior navigation service, especially in degraded signal environments. Compared with loosely and tightly coupled architectures, the Deep Integration (DI) architecture has better tracking and positioning performance. Information is shared among channels, and the assistant information from INS helps to reduce the dynamic stress of tracking loops. However, this vector tracking architecture may result in easy propagation of errors among tracking channels. To solve this problem, a Fault Detection and Exclusion (FDE) method for the deeply integrated BeiDou Navigation Satellite System (BDS)/INS navigation system is proposed in this paper. This method utilizes pre-filters’ outputs and integration filter’s estimations to form test statistics. These statistics can help to detect and exclude both step errors and Slowly Growing Errors (SGEs) correctly. The monitoring capability of the method was verified by a simulation which was based on a software receiver. The simulation results show that the proposed FDE method works effectively. Additionally, the method is convenient to be implemented in real-time applications because of its simplicity.

## 1. Introduction

The Chinese BeiDou Navigation Satellite System (BDS) plays an important role in GNSS, and it has been able to provide positioning and navigation service to global users since 28 December 2018 [1].

Position and velocity solutions based on GNSS are not subject to error accumulation by nature. However, in degraded signal environments, satellite signals are easily interrupted by building blocks, interference, and jamming [2]. For urban area navigation, the received signal intensity is typically 10–30 dB lower than the actual level of open environments [3]. In high dynamic scenarios, the bandwidth of the GNSS receiver is a tradeoff between dynamic adaptation and noise suppression [4]. Furthermore, the GNSS receiver usually cannot estimate the attitude of the user. In contrast, the INS is immune to interference and jamming. It has superior dynamic adaptation ability, and it supports attitude estimation. However, the INS is subject to error accumulation. All types of INS exhibit biases, scale factor and cross-coupling errors, and random noise to a certain extent. Therefore, the INS needs to be calibrated by the INS alignment and/or integration algorithms when it is used [5].

The integration of GNSS and INS can provide more accurate and reliable navigation information than either system alone, primarily in degraded signal environments. Traditional integration employs a loosely- or tightly-coupled architecture. The Deep Integration (DI) is promoted as having a superior performance to loose and tight coupling in scenarios with low C/N0 ratios. In a deeply integrated system, the INS can measure vehicle dynamics, which is fed into receiver tracking loops to reduce the tracking loop bandwidth and improve satellite signal acquisition ability. Furthermore, lowering the bandwidth results in greater noise suppression. In turn, the performance of the receiver is to be improved because of longer time integration and optimization of tracking loop parameters [6]. Edwards et al. described the implementation details of an embedded deeply integrated Global Positioning System (GPS)/INS software receiver on an FPGA platform [7]. A federated ultra-tightly coupled (UTC) algorithm based on pre-filters is proposed and the performance in high dynamic environments of the system is presented in [8]. The performance of a non-coherent deeply integrated navigation algorithm is compared with a tightly coupled navigation algorithm [9]. In [10], field tests of a UTC architecture with a Micro-Electro-Mechanical System (MEMS) IMU are performed both in indoor and outdoor environments.

There are mainly two deeply integrated architectures, named centralized filtering architecture and federated filtering architecture [5,11], respectively. Several DI varieties are compared in [6]. The centralized filter estimates INS and clock errors from I-Q integration values updated with high frequency and the dimension of the model is high. This architecture suffers from a heavy computation burden due to the complicated design, which is a problem for hardware implementation. In the federated architecture, the signals are firstly processed by a batch of pre-filters and then sent to the integration filter, which is updated with lower frequency. The dimension of the integration filter also decreases. The deep integration architecture proposed in this paper is a federated one based on the one in [12].

The GNSS receiver tracking structures can be classified into scalar tracking and vector tracking. In the scalar tracking loop, each channel only processes the input signal of its own. On the other hand, the vector tracking loop adjusts the Numerically Controlled Oscillator (NCO) by generating commands using the receiver’s velocity and position. In this approach, all the tracking channels share tracking information from each other, which is beneficial for strong signals assisting the weak signals’ reception. Hence, the vector tracking loop presents more superior performance than the scalar loo [6,13]. The deeply integrated BDS/INS navigation system whose NCO commands are generated from estimated or predicted solutions of the whole system has similar architecture and advantages with the vector tracking loop. While the vector tracking architecture provides an effective solution for dealing with situations in signal attenuation environments, it also allows easy propagation of errors among tracking loops [14]. The fault in a channel of the vector tracking system not only affects this channel’s measurements but also manifests itself in other channels’ measurements. This may easily cause the integrated filter to diverge.

The principle of Receiver Autonomous Integrity Monitoring (RAIM) is to use the least square or parity space vector algorithm to detect and exclude the faulty satellite using multiple redundancy measurements and obtaining the positioning integrity in time [15,16]. An algorithm detecting SGEs for a tightly integrated GPS/INS system which belongs to the scalar architecture is proposed in [17,18]. The authors suggested a rate detector based on the Autonomous Integrity Monitoring by Extrapolation Method (AIME) algorithm for SGE and a modification to the original algorithm is shown in [19]. There is extensive research on RAIM for the scalar tracking architecture [20,21,22], but it is unsuitable for application in vector tracking architecture because of easy propagation of errors among channels [23]. For deep integration and vector tracking architecture, few researchers concentrate on the identification and exclusion of fault channels. In [24], the pseudorange and pseudorange rate residuals computed from the code and carrier discriminators of every tracking loop are used as test statistics to analyze and determine interferences’ existence and which tracking channel is interfered. Zou et al. [25] proposed a novel robust algorithm based on a convolutional neural network (CNN) which can successfully suppress the fault propagation, but CNN is not suitable for the applications with the high real-time requirement.

A fault detection and exclusion method for the deeply integrated BDS/INS system is proposed in this paper. It can detect and exclude both step error and SGE effectively and it can be implemented in real-time application conveniently. A simulation test based on a software receiver was carried out for verification of RAIM algorithm monitoring ability. The remainder of this paper is organized as follows. Section 2 describes the framework of the deeply integrated BDS/INS system and the principles of pre-filters and integration filter. In Section 3, the fault detection and exclusion method for the integrated system with a vector loop architecture is introduced. Following this, the simulation setup and the performance results of the FDE method are provided in Section 4. Finally, in Section 5, concluding remarks and future work close this paper.

## 2. Deeply Integrated BDS/INS System

### 2.1. Implementation of DI BDS/INS System

**Figure 1:**
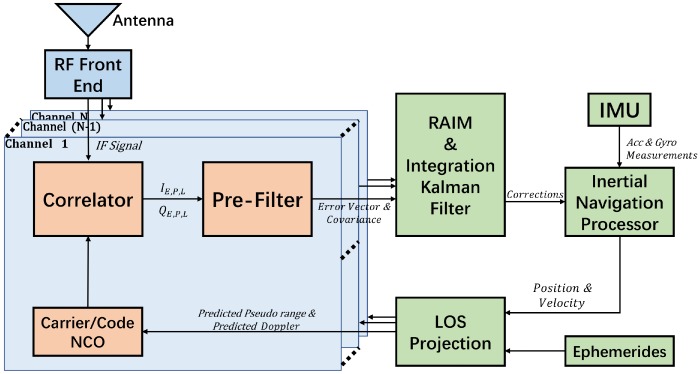
The framework of the deeply integrated BDS/INS navigation system.

The framework of the deeply integrated BDS/INS navigation system is presented in Figure 1.

In the deeply integrated BDS/INS System, as shown in this figure, the Intermediate Frequency (IF) signal from the Radio Frequency (RF) Front End is mixed with a local replica of the carrier to obtain in-phase and quadrature-phase signals. The signals are then transmitted to the receiver’s correlators, which can correlate in-phase and quadrature-phase signals with the one-half chip early, prompt, and one half chip late replica codes in *N* satellite tracking loop channels. The I-Q integration values of the correlators are further processed by pre-filters. The pre-filters can estimate the tracking errors and their covariances. Then, the outputs from all channels are sent to the RAIM and integration Kalman filter module. The RAIM module can identify and exclude faults in the satellite signal. The integration filter estimates a vector of error states which can periodically correct INS outputs, biases of the IMU and the clock bias and drift. The specific force and angular rate measurements collected by IMU are sent to the Inertial Navigation Processor to obtain high-frequency position and velocity estimates. Then, using Ephemerides, the receiver status is projected along the line of sight of each satellite to generate NCO commands, which control the generation of local carrier and code. Because all the satellite tracking channels share the dynamic information with each other and every satellite is integrated with the IMU, the tracking and positioning performance of the whole system is improved.

### 2.2. Pre-filters Based on Extended Kalman Filter (EKF)

There is one pre-filter for each channel to estimate tracking errors and their covariances. The structure of the pre-filter is based on the model described in [26]. The Intermediate Frequency (IF) signal for a visible BDS satellite can be modeled as follows [4]:

(1) $$\begin{array}{c} r\left(t\right)=A_{S}\cdot D\cdot C\left(t-\tau \right)\cdot \cos\left(2\pi \left(f_{I}F+f\right)t+\phi_{0}\right)+n\left(t\right) \end{array}$$

where $A_{s}$ represents the signal amplitude, *D* is the data modulation, *t* is time, *C* is the pseudorandom noise (PRN) code modulation, τ is the code delay in chips, $f_{I}F$ is IF in Hz, *f* is the carrier Doppler frequency in Hz, $\phi_{0}$ is the initial carrier phase, and *n* is additive white Gaussian noise.

After an integrate-and-dump operation, six baseband integral values $I_{E,P,L}$ and $Q_{E,P,L}$ can be obtained from the correlator outputs at the end of the integration interval. $I_{E,P,L}$ and $Q_{E,P,L}$ represent the early, prompt, and late In-phase/Quad-phase values, respectively.

(2) $$\begin{array}{cc} I_{m} & =A_{N}\cdot \frac{\sin(\frac{\delta \omega T}{2})}{\frac{\delta \omega T}{2}}\cdot R\left(\delta \tau +\Delta_{m}\right)\cdot \cos\left(\bar{\delta \phi }\right)+n_{I_{m}}\\ \; & =A\cdot R\left(\delta \tau +\Delta_{m}\right)\cdot \cos\left(\bar{\delta \phi }\right)+n_{I_{m}} \end{array}$$

(3) $$\begin{array}{cc} Q_{m} & =A_{N}\cdot \frac{\sin(\frac{\delta \omega T}{2})}{\frac{\delta \omega T}{2}}\cdot R\left(\delta \tau +\Delta_{m}\right)\cdot \sin\left(\bar{\delta \phi }\right)+n_{Q_{m}}\\ \; & =A\cdot R\left(\delta \tau +\Delta_{m}\right)\cdot \sin\left(\bar{\delta \phi }\right)+n_{Q_{m}} \end{array}$$

where m=E,PorL, $A_{N}$ is the signal amplitude, R(·) represents the code normalized correlation function, $\Delta_{E}=-d/2$, $\Delta_{P}=0$, $\Delta_{L}=+d/2$ (d is the time difference between the late and early correlators, usually 1 chip), *T* is the coherent integration time interval, and $n_{I_{m}}$ and $n_{Q_{m}}$ are the noise component of the In-phase and Quad-phase values. Since the attenuation due to frequency error is difficult to separate, the sin(x)/x term and $A_{N}$ are combined into *A*. $\bar{\delta \phi }$ is the average carrier phase error over the integration interval and it can be further expanded as [27,28]

(4) $$\begin{array}{c} \bar{\delta \phi }=\delta \theta +\delta \omega \frac{T}{2}+\delta \alpha \frac{T^{2}}{6} \end{array}$$

where δθ, δω, and δα are the carrier phase error, carrier frequency error, and carrier frequency error acceleration at the start of an integration interval, respectively.

The $I_{E,P,L}$ and $Q_{E,P,L}$ values are nonlinear functions of satellite signal parameters (Doppler, carrier phase, code delay, and so on); therefore, an EKF based tracking loop is implemented for each satellite. The $I_{E,P,L}$ and $Q_{E,P,L}$ values for each satellite at epoch *k* are incorporated into the tracking loop filter (pre-filter) as the measurement vector:

(5) $$\begin{array}{c} Z_{Pre,k}={\left[I_{E}\;\;Q_{E}\;\;I_{P}\;\;Q_{P}\;\;I_{L}\;\;Q_{L}\right]}_{k}^{T} \end{array}$$

where the subscript ${}^{\prime \prime }Pre^{\prime \prime }$ means pre-filters, while the superscript ${}^{\prime \prime }T^{\prime \prime }$ represents matrix transposition.

The signal parameter errors in the correlator outputs are used to update the measurements of the integration filter and the NCOs. Consequently, we estimate the errors of carrier and code instead of their “real” value. The state vector of the tracking loop filter at epoch *k* can be written as:

(6) $$\begin{array}{c} X_{Pre,k}={\left[\delta \theta \;\;\delta \omega \;\;\delta \alpha \;\;\delta \tau \;\;A\right]}_{k}^{T} \end{array}$$

where δθ is the carrier phase error (rad), δω is the carrier frequency error (rad/s), δα is the carrier frequency error acceleration ($rad/s^{2}$), δτ is the code phase error (chips), and *A* is the signal amplitude.

The state equation model of the Kalman filter for the BDS satellite signal tracking is given by [6,29]:

(7) $$\begin{array}{cc} X_{Pre,k+1} & =\Phi \cdot X_{Pre,k}+G\cdot W_{k}\\ \; & =\left[\begin{array}{ccccc} 1 & T & \frac{T^{2}}{2} & 0 & 0\\ 0 & 1 & T & 0 & 0\\ 0 & 0 & 1 & 0 & 0\\ 0 & \beta T & 0 & 1 & 0\\ 0 & 0 & 0 & 0 & 1 \end{array}\right]\cdot {\left[\begin{array}{c} \delta \theta \\ \delta \omega \\ \delta \alpha \\ \delta \tau \\ A \end{array}\right]}_{k}+\left[\begin{array}{ccccc} 1 & 0 & 0 & 0 & 0\\ 0 & 1 & 0 & 0 & 0\\ 0 & 0 & 1 & 0 & 0\\ 0 & 0 & \beta & 1 & 0\\ 0 & 0 & 0 & 0 & 1 \end{array}\right]\cdot {\left[\begin{array}{c} \omega_{clock}\\ \omega_{drift}\\ \omega_{accel}\\ \omega_{code}\\ \omega_{A} \end{array}\right]}_{k} \end{array}$$

where β is used to convert the units of radians to units of chips, while $W_{k}$ is the process noise vector for clock bias, clock drift, frequency rate error, code phase error, and the signal amplitude.

The nonlinear measurement equation, which is an abbreviated form of Equations (2) and (3), is defined as:

(8) $$\begin{array}{cc} Z_{Pre,k} & =h\left(X_{Pre,k}\right)+V_{Pre,k}\\ \; & \approx H_{Pre,k}\cdot X_{Pre,k}+V_{Pre,k} \end{array}$$

where *h* represents the nonlinear function of state variables, and the measurement noise vector $V_{Pre,k}$ consists of $n_{I_{m}}$ and $n_{Q_{m}}$ (m=E,PorL). $H_{Pre,k}$ is the sensitivity matrix of $h(X_{Pre,k})$, which is defined as:

(9) $$\begin{array}{c} H_{Pre,k}=\frac{\partial h(X_{Pre,k})}{\partial X_{Pre,k}} \end{array}$$

The noise variance of the measurement vector is computed as a function of the carrier-to-noise ($C/N_{0}$) [6]:

(10) $$\begin{array}{c} \sigma_{n_{I}}^{2}=\sigma_{n_{Q}}^{2}=\frac{1}{2\cdot 10^{0.1C/N_{0}}\cdot T} \end{array}$$

where $n_{I}$ and $n_{Q}$ are the noise of *I* and *Q* prompt values, respectively.

### 2.3. BDS/INS Integration Kalman Filter

The integrated BDS/INS system is based on a 17-state EKF, which is based on the first-order linearization on the nonlinear system model with the assumption of Gaussian distributed noises. The components of the state vector $X_{Nav,k}$ are defined as follows and described in Table 1. The subscript ${}^{\prime \prime }Nav^{\prime \prime }$ stands for navigation.

(11) $$\begin{array}{c} X_{Nav,k}={\left[\delta \varphi \;\;\;\delta v^{n}\;\;\;\delta p\;\;\;\varepsilon^{b}\;\;\;\nabla^{b}\;\;\;b_{clk}\;\;\;d_{clk}\right]}_{k}^{T} \end{array}$$

The attitude and velocity are resolved in the local navigation frame and they are Earth-referenced. The superscript *n* represents the local navigation frame and the superscript *b* represents the body frame. The position error is expressed in terms of the latitude *L*, longitude λ, and height *h*, respectively:

(12) $$\begin{array}{c} \delta p={\left[\delta L\;\;\;\delta \lambda \;\;\;\delta h\right]}^{T} \end{array}$$

The propagation of the errors can be obtained from a set of difference equations. The discrete-time state transition matrix $\Phi_{Nav,k}$ and error dynamics are derived from [5,30]. Thus, the linearized system propagation equation is expressed as

(13) $$\begin{array}{cc} X_{Nav,k+1} & =\Phi_{Nav,k}\cdot X_{Nav,k}+W_{Nav,k} \end{array}$$

where $W_{Nav,k}$ is the system noise vector. The measurement equation could be given as follows:

(14) $$\begin{array}{cc} Z_{Nav,k} & =H_{Nav,k}\cdot X_{Nav,k}+V_{Nav,k} \end{array}$$

where $V_{Nav,k}$ is the measurement noise vector. The outputs of the pre-filters are taken as measurements for the integration filter update. Since the NCO commands are generated using the INS status information, the tracking errors estimated by the pre-filters have relationships with the residual errors of the INS. The measurement vector $Z_{Nav}$ at epoch *k* of the integration filter can be defined as:

(15) $$\begin{array}{cc} Z_{Nav,k} & ={\left[\delta \rho ,\delta \dot{\rho }\right]}_{k}^{T}\\ \; & ={\left[\delta \rho_{1},\delta \rho_{2},\ldots ,\delta \rho_{N},\delta {\dot{\rho }}_{1},\delta {\dot{\rho }}_{2},\ldots ,\delta {\dot{\rho }}_{N}\right]}_{k}^{T} \end{array}$$

The pseudorange error $\delta \rho_{i}$ and the pseudorange rate error $\delta {\dot{\rho }}_{i}\left(i=1,2,\ldots ,N\right)$ are obtained from code phase error and carrier frequency error estimated by pre-filters, respectively. The transformation is shown in Equations (16) and (17).

(16) $$\begin{array}{c} \delta \rho_{i}=\frac{c}{f_{code}}\delta \tau_{i} \end{array}$$

(17) $$\begin{array}{c} \delta {\dot{\rho }}_{i}=\frac{c}{2\pi f_{carr}}\delta \omega_{i} \end{array}$$

where *i* means the *i*th satellite tracking channel of all N channels. The symbol *c* is the speed of light. $f_{code}$ represents the code frequency and $f_{carr}$ represents the carrier frequency. δτ and δω are defined in Equation (6). The noise covariance matrix $R_{Nav,k}$ of the measurement vector is directly related to the pre-filter’s state covariance matrix and more details can be found in [12].

The observation matrix $H_{Nav}$ at epoch *k* is given in Equation (18). It is linearized to accommodate the measurement vector of the integration filter.

(18) $$\begin{array}{c} H_{Nav,k}={\left[\begin{array}{cccc} O_{N\times 6} & H_{\rho 1} & O_{N\times 6} & H_{\rho 2}\\ O_{N\times 3} & H_{\dot{\rho }1} & O_{N\times 9} & H_{\dot{\rho }2} \end{array}\right]}_{k} \end{array}$$

where $O_{M\times N}$ is a zero matrix of size M×N. $H_{\rho 1}$, $H_{\rho 2}$, $H_{\dot{\rho }1}$, and $H_{\dot{\rho }2}$ are defined as follows:

(19) $$\begin{array}{cc} H_{\rho 1} & =-\left[\begin{array}{c} u_{1}\\ u_{2}\\ \vdots \\ u_{N} \end{array}\right]\cdot C_{1},H_{\rho 2}=\left[\begin{array}{c} 1_{N\times 1}\;\;O_{N\times 1} \end{array}\right] \end{array}$$

(20) $$\begin{array}{cc} H_{\dot{\rho }1} & =-\left[\begin{array}{c} u_{1}\\ u_{2}\\ \vdots \\ u_{N} \end{array}\right]\cdot C_{2},H_{\dot{\rho }2}=\left[\begin{array}{c} O_{N\times 1}\;\;1_{N\times 1} \end{array}\right] \end{array}$$

(21) $$\begin{array}{cc} C_{1} & =\left[\begin{array}{ccc} -(R+h)\cos\lambda \sin L & -(R+h)\cos L\sin\lambda & \cos L\cos\lambda \\ -(R+h)\sin\lambda \sin L & (R+h)\cos L\cos\lambda & \cos L\sin\lambda \\ R(1-e^{2})+h & 0 & \sin L \end{array}\right] \end{array}$$

(22) $$\begin{array}{c} C_{2}=\left[\begin{array}{ccc} -\sin\lambda & -\sin L\cos\lambda & \cos L\cos\lambda \\ \cos\lambda & -\sin L\sin\lambda & \cos L\sin\lambda \\ 0 & \cos L & \sin L \end{array}\right] \end{array}$$

where $u_{i}$ is the unit vector of the line-of-sight direction from the user navigation solution to the *i*th satellite. $C_{1}$ and $C_{2}$ are coordinate transformation matrixes of different frames. $1_{M\times N}$ is an M×N matrix whose elements are all 1s. *R* is the radius of curvature in prime vertical and *e* is the primary eccentricity of the ellipsoid of the Earth’s surface.

## 3. Fault Detection and Exclusion Method

It is important to maintain reliability and stability for the deeply integrated system. RAIM is a receiver-based autonomous integrity method to ensure the smooth running of the whole system and provide a timely warning to users when the positioning is not reliable. The process of RAIM usually contains two main steps. The first step is fault detection and exclusion. It typically uses multiple redundancy measurements to check the consistency. The second step is calculating Protection Level (PL). In this paper, we only concentrate on the first step.

**Figure 2:**
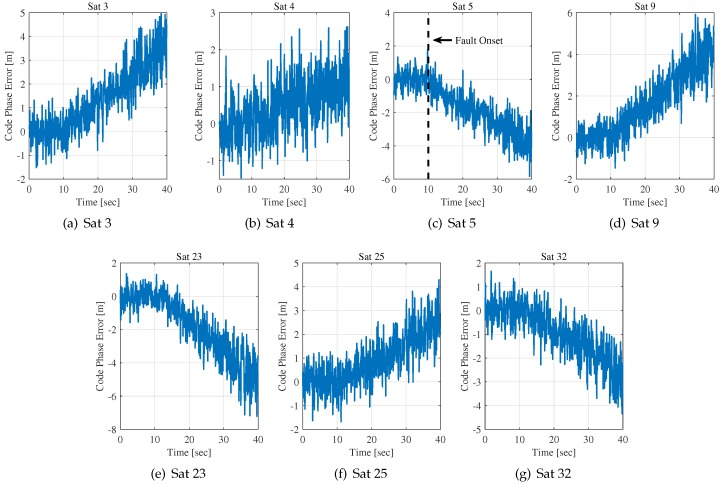
Propagation of errors among channels of the DI BDS/INS system.

RAIM for the conventional scalar tracking is not suitable for the deeply integrated BDS/INS system, especially for fault exclusion. The vector tracking architecture of the system brings not only performance improvement but also easy propagation among channels. As a result, the pseudorange measurements are contaminated by the faulty channel. A simulation was performed to validate this. As shown in Figure 2, a slowly growing error of slope 1 m/s in satellite 5 is injected into the IF signals at t=10 s. The code phase error estimations obtained from pre-filter outputs are plotted in this figure.

The figure shows seven satellite channels’ code tracking error information. It is obvious that, after the fault injection into one channel’s signal, the average absolute values of other six channels’ code phase errors more or less increase with the fault’s amplitude.

It should be noted that the RAIM methods for the scalar loop and the tightly-coupled system typically use pseudorange residuals computed by subtracting predicted or estimated pseudorange from pseudorange measurement for integrity monitoring. Nevertheless, the pseudorange residuals mentioned above are not suitable for fault detection of the vector loop RAIM algorithms [31]. The corrupted pseudorange measurements due to propagation of fault violate the scalar loop RAIM assumption that faulty channels should be less than N−L [32], where *L* is the number of states to be estimated. As discussed in [14], the code phase error estimations of vector loop have a similar mathematical model with the pseudorange residuals of the scalar loop which can be used to form statistics for integrity monitoring. They can be modeled in meters at epoch *k* as follows:

(23) $$\begin{array}{cc} \delta \rho_{k} & =H_{P,k}\left(X_{P,k}-X_{P,k}^{-}\right)+\epsilon_{k}\\ \; & =H_{P,k}\Delta X_{P,k}+\epsilon_{k} \end{array}$$

(24) $$\begin{array}{cc} or\;\;\epsilon_{k} & =\delta \rho_{k}-H_{P,k}\Delta X_{P,k} \end{array}$$

where the definition of $\delta \rho_{k}$ can be found in Equations (15) and (16). $X_{P,k}$ is a vector of the real receiver position and clock bias and the subscript ${}^{\prime \prime }P^{\prime \prime }$ means positioning related. $X_{P,k}^{-}$ is the priori estimation of $X_{P,k}$ extracted from INS and clock status. $\Delta X_{P,k}$ represents the error state vector of the prior estimation $X_{P,k}^{-}$, and $H_{P,k}$ is the pseudorange conversion matrix. $\epsilon_{k}$ is additive Gaussian noise. Ephemeris errors, satellite clock errors, and atmospheric delay errors are not modeled in detail and they will be explored in future work. $X_{P,k}$ and $H_{P,k}$ are defined below:

(25) $$\begin{array}{c} X_{P,k}={\left[x\;\;y\;\;z\;\;b_{clk}\right]}_{k}^{T} \end{array}$$

(26) $$\begin{array}{c} H_{P,k}=\left[\begin{array}{cc} -u_{1} & 1\\ -u_{2} & 1\\ \vdots & \vdots \\ -u_{N} & 1 \end{array}\right] \end{array}$$

where ${\left[x\;\;y\;\;z\right]}^{T}$ are receiver coordinate in Earth-Centered Earth-Fixed (ECEF) frame and $b_{clk}$ is the clock bias. ${\left[u_{1},u_{2},\ldots ,u_{N}\right]}^{T}$ is defined below Equation (19).

In this paper, we only consider the single fault scenario and two types of errors are taken into account. One is the abrupt step error whose magnitude remains constant and another is SGE, which grows with time. The fault vector $f_{k}$ injected into a single channel can be modeled as:

(27) $$\begin{array}{cc} f_{k} & ={\left[0,0,\cdots ,f_{i},\cdots ,0\right]}_{K}^{T}\;\;\;\left(i=1,2,\cdots ,N\right) \end{array}$$

(28) $$\begin{array}{cc} f_{i} & =a\cdot \Delta t+b\;\;\;(a=0,\;b\neq 0\;or\;a\neq 0,\;b=0) \end{array}$$

where $f_{k}$ is an N×1 vector and $f_{i}$ is the fault amplitude of the selected faulty channel’s signal. *a* is the slope of the SGE and *b* is the amplitude of the abrupt step error. Δt means the time difference between the epoch where SGE starts and epoch *k*. Once a fault occurs in one channel, the code phase errors of all the vector tracking channels will change. In such circumstance, derived from Equation (23), the code phase error estimations from pre-filters’ outputs can be given as:

(29) $$\begin{array}{c} \delta \rho_{k}=H_{P,k}\Delta X_{P,k}+f_{k}+\epsilon_{k} \end{array}$$

or it can be expressed as the noise term $r_{k}$ of the pseudorange error estimations:

(30) $$\begin{array}{c} r_{k}=\delta \rho_{k}-H_{P,k}\Delta X_{P,k}=f_{k}+\epsilon_{k} \end{array}$$

As illustrated in Figure 2, the term $H_{P,k}\Delta X_{P,k}+f_{k}$ represents the mean values of the code phase error estimations. The mean values of all channels’ estimations begin to change after t=4 s and they look the same, but there is a distinction between the faulty channel and the contaminated channels. For the faulty channel (Sat 5), the element $f_{i}$ is non-zero and increases with time. $H_{P,k}\Delta X_{P,k}$ and $f_{k}$ contribute to the change of the mean value of estimations together. On the other hand, the element $f_{i}$ for other channels is zero. It means that changes in the mean values of other channels are only caused by the term $H_{P,k}\Delta X_{P,k}$.

$\Delta X_{P,k}$ is the vector of position error and clock bias error between receiver real position and a prior estimation. Assuming that the deeply integrated system is aligned and the receiver works steadily, when there is no fault in the satellite signals, the expectation value of the term $\Delta X_{P,k}$ should be zero and $\Delta X_{P,k}$ is generally small. On the other hand, the measurement update process of the deeply integrated navigation filter estimates corrections for the INS and clock status. The corrections updated every fixed interval for the position and clock bias amendment accumulate into the term $\Delta X_{P,k}$. Hence, the expectation value of the corrections’ accumulation is zero, too. Once a fault appears in the signal, the filter will be polluted and the corrections estimated after that will be led into the opposite direction, which means the minus sum of the corrections can be regarded as an estimation for the term $\Delta X_{P,k}$. It can be described as:

(31) $$\begin{array}{c} \hat{\Delta X_{P,k}}=-\sum_{j=k-(M-1)}^{k}\Delta X_{Index,j}^{+} \end{array}$$

where $\hat{\Delta X_{P,k}}$ is the estimation of the term $\Delta X_{P,k}$. $\Delta X_{Index,k}^{+}$ is a subset of the state vector estimation after measurement update of the integrated filter at epoch *k* and it is expressed in the ECEF coordinate. *M* is the accumulation window length. The subscript ${}^{\prime \prime }Index^{\prime \prime }$ indicates this term is extracted and the index numbers, which can be found in Table 1, are 7, 8, 9, and 16. The superscript ${}^{\prime \prime }{+}^{\prime \prime }$ stands for posterior estimation.

In this paper, we adopt the weighted RAIM algorithm introduced in [20] for integrity monitoring. It is a snapshot fault detection method, which means that the test statistic to flag fault only depends on the measurement residuals of the present epoch. The test statistic *s* at epoch *k* is defined as the square root of the Weighted Sum of the Squared Errors (WSSE). WSSE at epoch k is described as follows:

(32) $$\begin{array}{cc} WSSE_{k}=s_{k}^{2} & ={r_{k}}^{T}W_{k}r_{k}\\ \; & ={\left(\delta \rho_{k}-H_{P,k}\Delta X_{P,k}\right)}^{T}W_{k}\left(\delta \rho_{k}-H_{P,k}\Delta X_{P,k}\right) \end{array}$$

where $W_{k}$ is the weight matrix related to the standard deviation of the noise term $\epsilon_{k}$. $W_{k}$ is given by the inversion of the covariance matrix $R_{\rho ,k}$, which is a diagonal matrix. $R_{\rho ,k}$ is the pseudorange corresponding part of $R_{Nav,k}$ mentioned above. Therefore, $W_{k}$ can be computed as:

(33) $$\begin{array}{c} W_{k}={\left(E\left\{\epsilon_{k}\epsilon_{k}^{T}\right\}\right)}^{-1}=R_{\rho ,k}^{-1} \end{array}$$

Substituting Equations (31) and (33) into Equation (32), we can get the square of the statistic *s* at epoch *k*:

(34) $$\begin{array}{c} s_{k}^{2}={\left(\delta \rho_{k}+H_{P,k}\sum_{j=k-(M-1)}^{k}\Delta X_{Index,j}^{+}\right)}^{T}R_{\rho ,k}^{-1}\left(\delta \rho_{k}+H_{P,k}\sum_{j=k-(M-1)}^{k}\Delta X_{Index,j}^{+}\right) \end{array}$$

**Figure 3:**
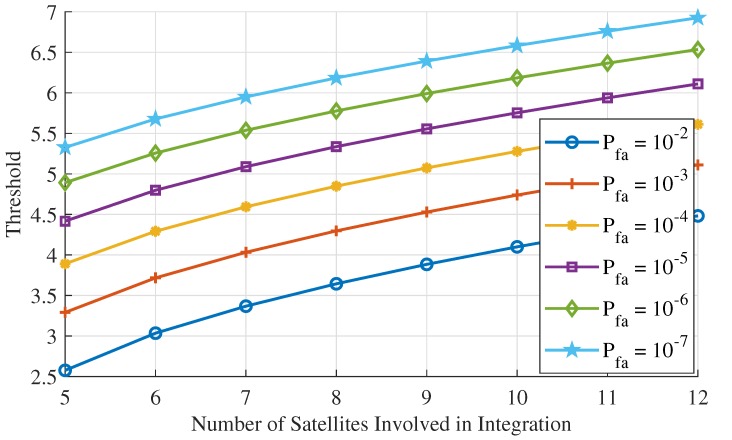
Threshold $T_{th}$ for different $P_{fa}$ and satellite number *N*.

The fault detection test is a binary hypothesis test. At the epoch *k*, if the statistic *s* is below the threshold $T_{th}$, which is a prepared constant, the pseudorange error estimations are considered reliable. On the other hand, if the statistic exceeds the threshold, they are assumed as unsafe. Under fault-free conditions, $s_{k}^{2}$ obeys a chi-squared distribution with the freedom of N−4 degrees. Once a fault with a magnitude of $f_{i}$ occurs in one channel’s signal, $s_{k}^{2}$ will be a noncentral chi-squared distribution with the freedom of N−4. Under normal conditions, the threshold $T_{th}$ can be selected analytically [20]. It is a function of the probability of false alarms ($P_{fa}$) and the number of visible satellites (*N*). Given the $P_{fa}$, the $T_{th}$ can be calculated by inverting the incomplete gamma function:

(35) $$\begin{array}{c} 1-P_{fa}=\frac{1}{2^{a}\Gamma \left(a\right)}\int_{0}^{T_{th}^{2}}e^{-s/2}s^{a-1}ds \end{array}$$

where a=(n−4)/2, Γ is the Gamma function. The values can be computed and stored beforehand for fault detection, and several sets of $T_{th}$ for different *N* and $P_{fa}$ are plotted in Figure 3.

For fault exclusion, a w-test method is applied. The fault exclusion statistic for the ith satellite channel is constructed as:

(36) $$\begin{array}{cc} w_{i,k} & =\left|\frac{-e_{i}^{T}r_{k}}{\sqrt{e_{i}^{T}R_{\rho ,k}e_{i}}}\right| \end{array}$$

(37) $$\begin{array}{cc} r_{\rho ,k} & =\delta \rho_{k}+H_{P,k}\sum_{j=k-(M-1)}^{k}\Delta X_{Index,j}^{+} \end{array}$$

where $e_{i}={\left[0,0,\ldots 0,1,0,\ldots ,0\right]}^{T}$ whose the *i*th element is 1 and the others are 0. When a fault occurs and it is detected by WSSE test at epoch *k*, the statistic $w_{i,k}$ will be calculated for every channel. The maximum value among them is regarded as the faulty channel’s statistic and this channel should be excluded from the integration measurement set.

## 4. Simulation and Results

A simulation test was carried out to validate the proposed FDE method. This test was implemented on a Matlab-based software receiver. It would be complex to analyze FDE method with real data because faults rarely occur in real data and they are hard to grasp. Therefore, simulation studies were adopted here, and they can help to get better insights into the ability of the monitoring algorithm. Additionally, the fault should be injected in IF signals rather than pseudorange measurements because of the vector tracking architecture. Otherwise, the simulation test is neither effective nor convincing.

Before showing the simulations and results, a summary of the assumptions is made to develop the fault detection and exclusion method for the deeply integrated BDS/INS system. Further research will be conducted in future work.

- The receiver noise in the simulations had negligible residuals such as ephemeris errors, satellite clock errors, atmospheric delay errors, and multipath errors. IF signals were generated with standard atmospheric models.
- For INS simulation, only constant bias and random walk noise were modeled. Scale factor errors, askew installation errors, correlated bias errors, and lever arm errors were not modeled in the simulation.
- The DI system was calibrated properly and it worked steadily before the fault occurs.

### 4.1. Simulation Setup

**Figure 4:**
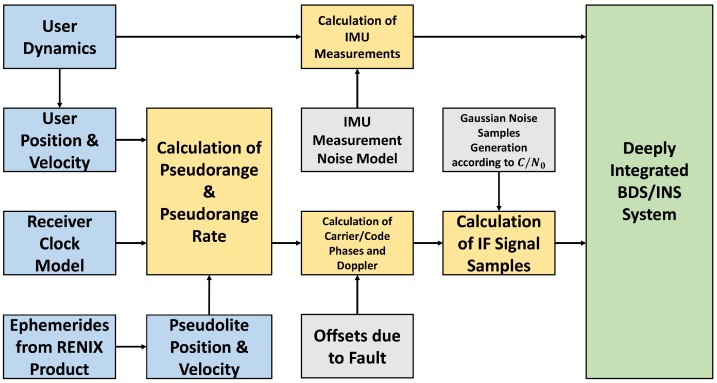
The framework of the simulation scheme based on Matlab.

The framework of simulation is shown is Figure 4. The figure mainly describes the generation approach of IF signals and IMU measurements. Moreover, the functions of the framework’s modules and their relationships are illustrated. Noise generation and Fault injection are expressed in gray boxes.

**Figure 5:**
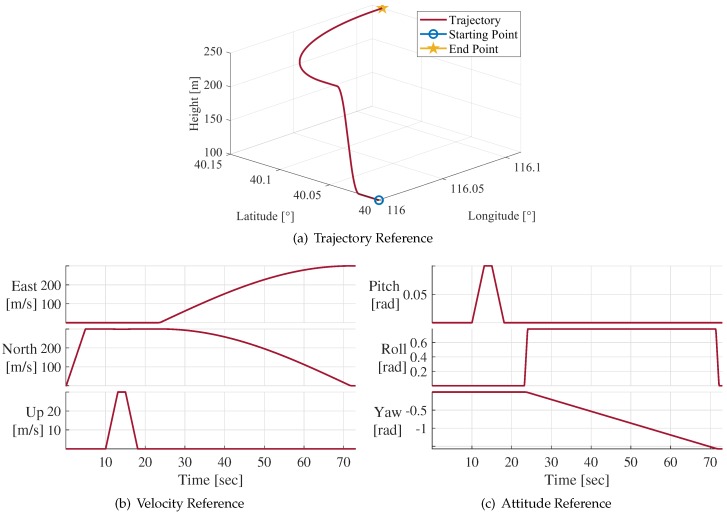
Receiver trajectory, velocity, and attitude reference.

In this simulation, IF signal samples were generated according to the receiver trajectory designed in advance. The starting point coordinate was set as ”$40^{\circ }N,116^{\circ }E$” and the altitude was 100 m. The movement states of the receiver included acceleration, uniform moving, climbing, and turning. To reveal the receiver movement more apparently, the receiver trajectory, velocity, and attitude references are plotted in Figure 5a–c, respectively.

The IMU measurements without noise were calculated according to the trajectory, velocity, and attitude references of the receiver. Then, we added artificial IMU bias and random walk noise to the raw measurements. Finally, the IMU measurements were stored and prepared for later processing. The noise parameters were designed in accordance with the MEMS grade IMU [33]. Table 2 shows the IMU noise parameters and sample rate.

**Figure 6:**
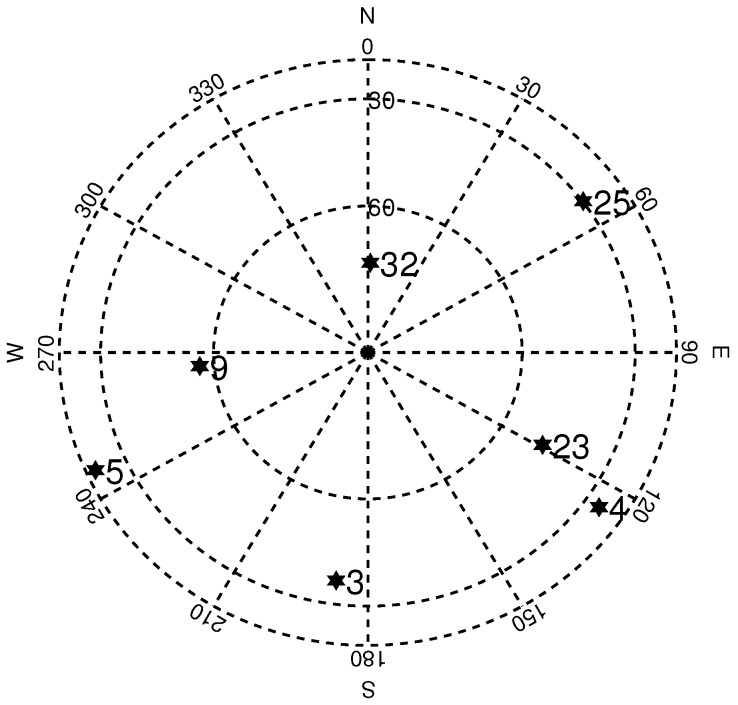
BDS satellite visibility in simulation.

Pseudolite model was applied in this simulation scenario. Actual BDS satellite ephemerides were achieved from International GNSS Service (IGS) products for pseudolite simulation. The broadcast ephemerides were collected on 1 February 2020 from BCEmerge. The BDS satellite orbits and movements were simulated using the ephemerides mentioned above. Through this approach, the position and velocity of BDS satellites could be calculated for IF signal generation. The DI system utilizes the same ephemerides for positioning and integration. We selected seven of the visible BDS satellites in the ephemerides for this simulation. The sky plot of the seven BDS satellites at the starting epoch is shown in Figure 6.

With the dynamic information of users and satellites, the line-of-sight (LOS) range and doppler could be computed. Clock bias, clock drift, and atmospheric delay generated with standard atmospheric models were added to get pseudorange and pseudorange rate. The carrier phase and code phase were then calculated for IF signal samples. The fault for RAIM monitoring algorithm validation was added into the code phase in this step. According to the BDS B3I signal format, the IF signals’ parameters were set as follows. The carrier frequency was 1268.52 MHz and the PRN code frequency was 10.23 MHz. The sampling frequency for IF signals was 25 MHz. A random sequence only including −1 and +1 at a rate of 50 Hz was used for bit modulation. Next, artificial white Gaussian noise was added to the digital IF data based on the $C/N_{0}$ value predefined. The digital IF data have a $C/N_{0}$ value of 44 dB-Hz for all satellites. Finally, the digital IF data were quantized and stored in a text file.

A software-defined DI BDS/INS system based on Matlab processed the IF signals and IMU measurements to valid the RAIM algorithm monitoring capability. The simulation results are presented next.

### 4.2. Simulation Results

In this simulation, two types of faults including step error and slowly growing error were injected in IF signals. RAIM algorithm performance was evaluated by fault detection time from when the fault was onset. The alteration of statistics and noise are presented and analyzed. The probability of false alarm $P_{fa}$ is $10^{-5}$/h and the satellite number *N* is 7, thus the threshold $T_{th}$ for fault detection is supposed to be 5.089, as shown in Figure 3.

#### 4.2.1. Step Error Simulation

**Figure 7:**
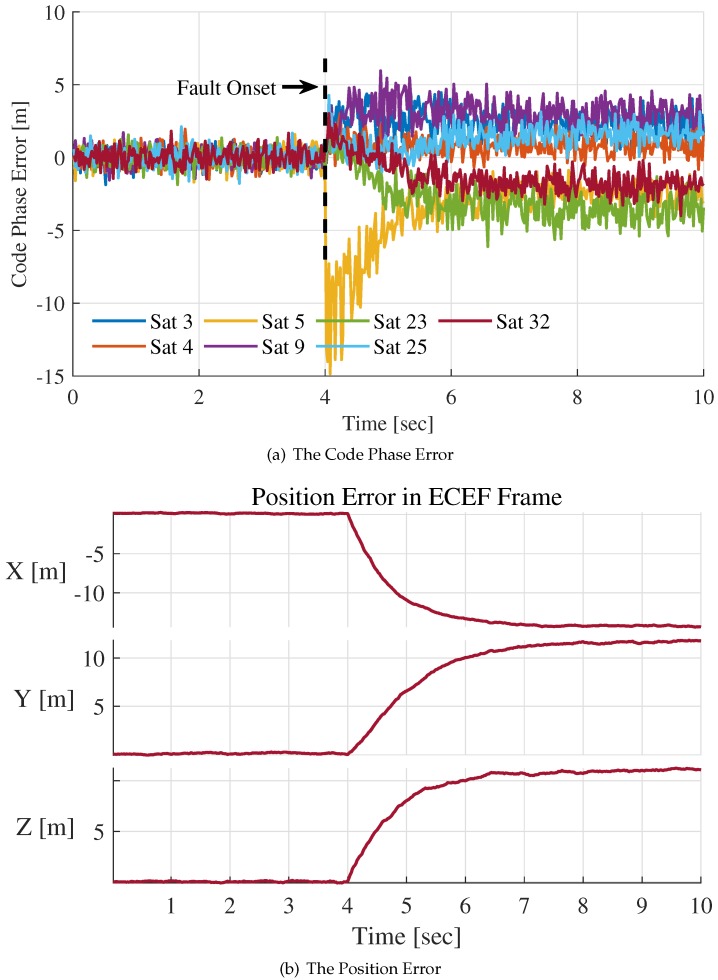
The position error and code phase error estimation for step fault of 20 m.

We added a step fault of 20 m at 4 s in the PRN code phase during the IF signal generation process. In Figure 7, the changes of code phase error estimations and position errors are presented. The code phase error estimations for all channels were obtained from pre-filters’ outputs at 50 Hz. The position error is the difference between INS positioning estimations and trajectory reference.

After the fault appearance at t=4 s, the code phase error estimation of Satellite 5 has an abrupt change. Then, the code phase error decreases and tends towards stability, as shown in Figure 7a. The reason the code phase error estimation of Satellite 5 at t=4 s is about −15 m rather than −20 m is that pre-filer has inertial and smooth properties. The rapid fault propagation among channels can be observed after t=4 s. The code phase error estimations of contaminated channels float more or less.

The position error is presented in ECEF frame, as shown in Figure 7b. The propagation of errors is mainly aroused by position error since NCO command generation of all channels uses the same positioning result estimated by the INS. Therefore, the trend of fault propagation is consistent with the trend of positioning error, which can be watched in Figure 7a,b.

**Figure 8:**
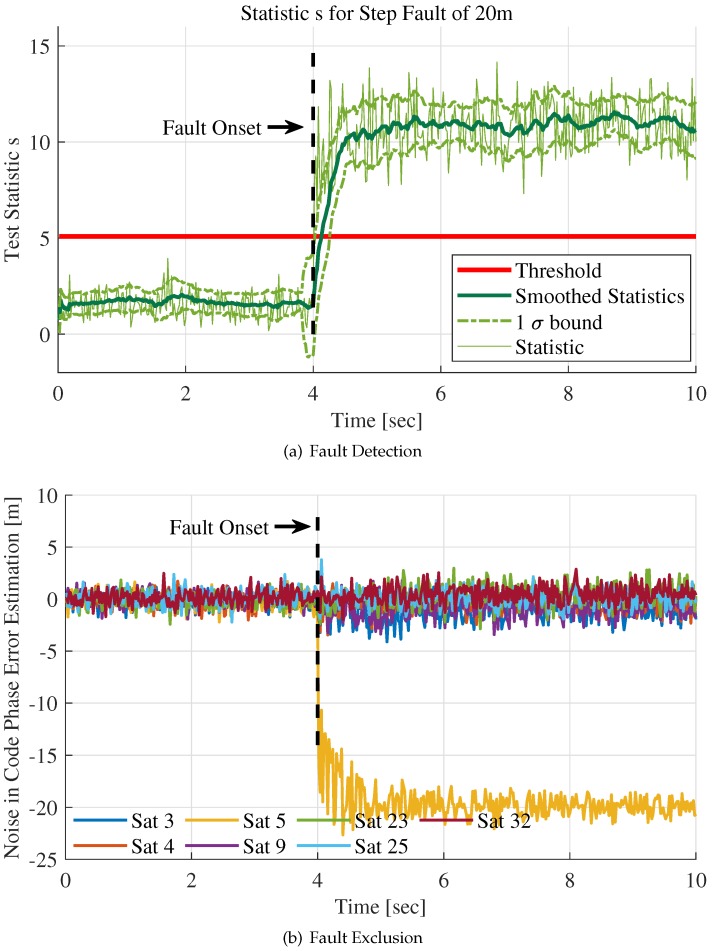
Fault detection and exclusion for step fault of 20m.

Figure 8 shows the test statistics over time for both fault detection and exclusion.

For fault detection, the test statistic defined in Equation (34) is applied. The test statistic *s* in Figure 8a bumps up and exceeds the threshold $T_{th}$ at t=4 s where the step fault occurs. It reveals that the RAIM algorithm can detect the fault immediately. The mean and 1-σ bound of the statistic *s* over time is also plotted in Figure 8a. They are calculated by using a sliding window including 10 statistic samples. The mean and standard deviation values of all sliding windows are plotted with a thicker green line in this figure. On account of the abrupt increase of the statistic, *s* at t=4 s, the 1-σ bound fluctuates fiercely at the same time.

To contrast the faulty channel’s noise with the counterparts of contaminated channels, the statistic $r_{\rho ,k}$ defined in Equation (30) is plotted in Figure 8b for fault exclusion. The term $r_{\rho ,k}$ consists of $f_{k}$ and $\epsilon_{k}$. $f_{k}$ is only non-zero for the faulty channel ${}^{\prime \prime }Sat5^{\prime \prime }$ and $\epsilon_{k}$ has almost same noise deviation for all channels. As can be seen in Figure 8b, the components of the term $r_{\rho ,k}$, which stands for the noise in code phase error estimations, show striking differences between the faulty channel and contaminated channels after the fault emergence. The component for ${}^{\prime \prime }Sat5^{\prime \prime }$ decreases abruptly at t=4 s and then fluctuates around −20 m, which is the same as the fault amplitude. It reveals that the statistic $r_{\rho ,k}$ excludes the faulty channel successfully.

**Figure 9:**
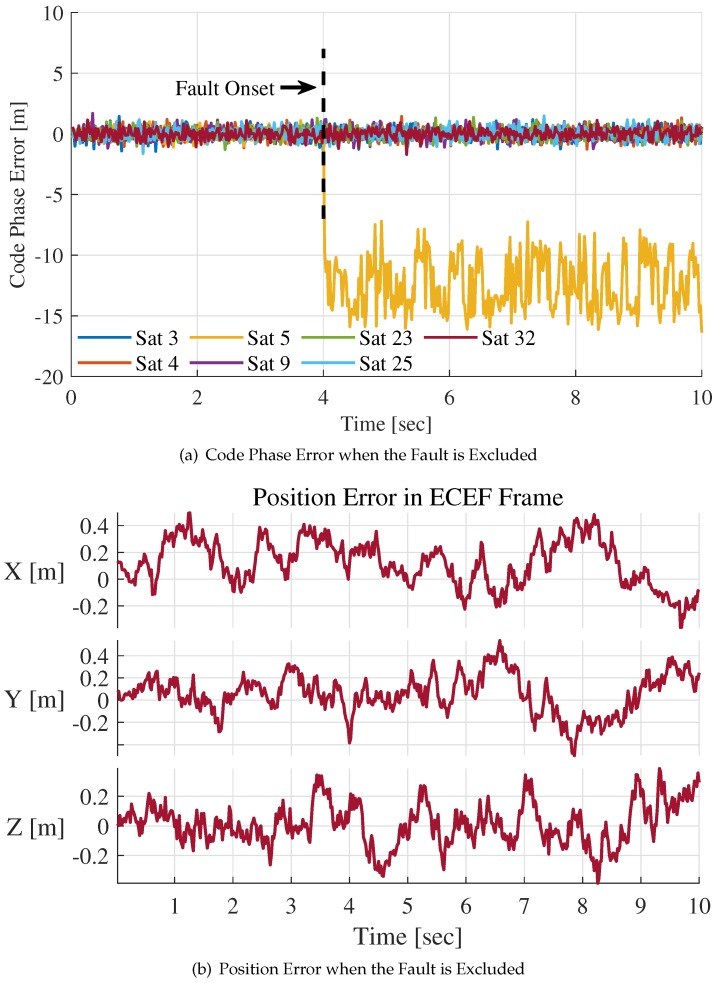
Code phase error estimations and position error for step fault of 20 m after fault exclusion.

To express the changes of test statistic explicitly, the faulty channel is not isolated during the integration process which is illustrated by above figures. Figure 9a,b indicates the situation when the fault is detected and excluded successfully with the proposed method.

In Figure 9a, after the fault is excluded at t=4 s, the code phase error estimations of other channels are no longer contaminated. The faulty channel ${}^{\prime \prime }Sat5^{\prime \prime }$ still maintains tracking state because of vector tracking architecture. The reason the mean value of the code phase error estimation of ${}^{\prime \prime }Sat5^{\prime \prime }$ is not −20 m is that the pre-filter’s measuring range is ±15 m. Additionally, the signal is tracked at a low carrier-to-noise ratio. Figure 9b shows the position error of the system when the fault is isolated. We can conclude that the positioning results are not affected by the 20 m step fault with the help of the proposed method.

#### 4.2.2. Slowly Growing Error Simulation

**Figure 10:**
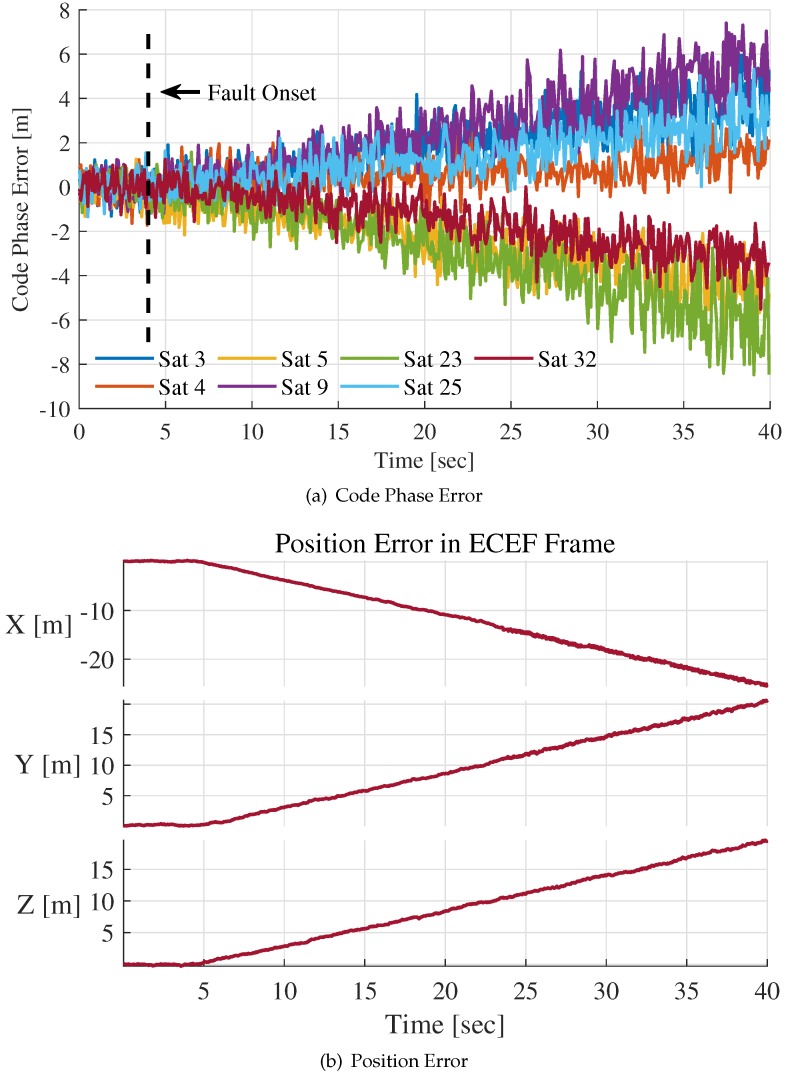
Code phase error estimations and position error for 1 m/s SGE fault.

In this part, the simulation for SGE is performed to valid the RAIM algorithm monitoring ability. Figure 10a,b show code phase error estimations and the position error when a 1 m/s SGE fault is injected in ${}^{\prime \prime }Sat5^{\prime \prime }$ at t=4 s.

Error propagation among channels can be observed since the 1 m/s SGE fault injection at t=4 s. The absolute value of some contaminated channel’s code phase error is even larger than that of the faulty channel ${}^{\prime \prime }Sat5^{\prime \prime }$. It is hard to distinguish the faulty channel from all channels involved in integration by code phase error estimations only. The absolute values of code phase error estimations for all channels increase with the growth of position error. Although we are unable to know exact position error in practical application, the corrections estimations (Equation (31)) influenced by the SGE fault can help to establish the statistics that are defined in Equations (34) and (37) for fault detection and exclusion.

To test the RAIM method monitoring capability effectively, two groups of Monte Carlo runs were simulated for 1m/s SGE and 0.5m/s SGE, respectively. Thirty test simulation tests were carried out for each group. The fault detection times since the fault injection were recorded and analyzed. Table 3 summarizes the mean and standard deviation of the fault detection times.

**Figure 11:**
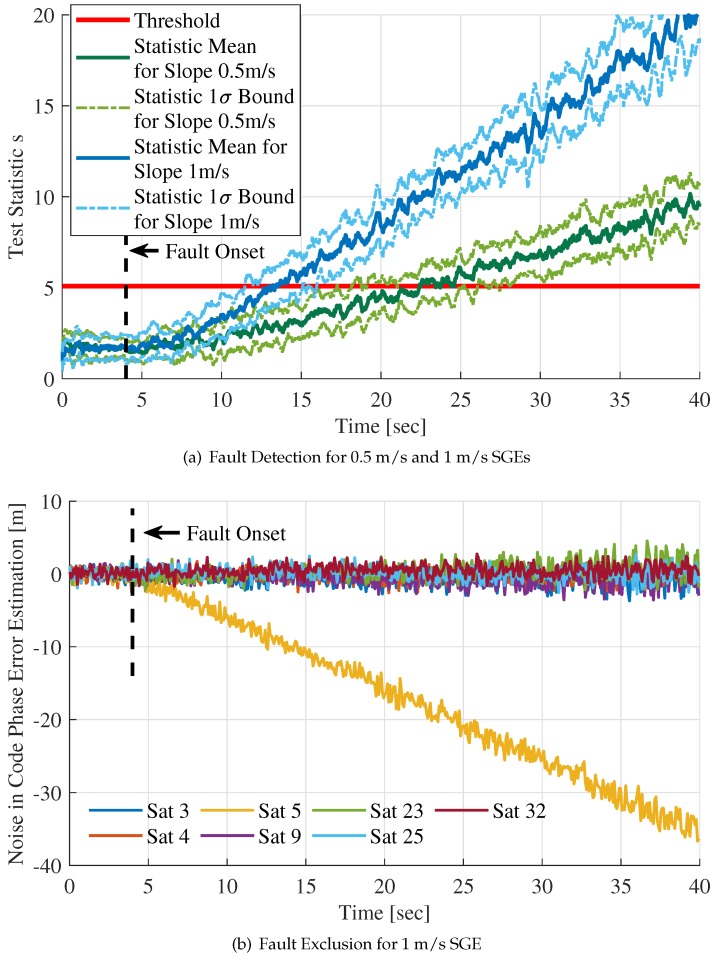
Fault detection and exclusion for 0.5 m/s and 1 m/s slowly growing errors.

The test results of one sample in each Monte Carlo group are shown in Figure 11. In Figure 11a, the mean and 1-σ bound of the statistic *s* over time for both 0.5 m/s SGE and 1 m/s SGE are presented. The statistics for 0.5 m/s SGE and 1 m/s SGE exceed the threshold $T_{th}$ at t=22.5 s and t=12.7 s, respectively. The fault detection time can be computed as 18.5 s and 8.7 s. A statistic rate detector algorithm referenced in [19] can be utilized here to reduce detection time and promote the sensitivity of fault detection. It will be researched in future work.

Figure 11b shows the statistic $r_{\rho ,k}$ for 1 m/s SGE fault exclusion. Because the statistic $r_{\rho ,k}$ for 0.5 m/s SGE has similar changing trend with 1 m/s SGE, it is not plotted in this figure in order to observe $r_{\rho ,k}$ more explicitly. The component of statistic $r_{\rho ,k}$ for faulty channel ${}^{\prime \prime }Sat5^{\prime \prime }$ decreases with the rise of SGE amplitude. The w-test statistic of the faulty channel is the largest and it is easily distinguished from that of contaminated channels when statistic *s* exceeds the threshold.

**Figure 12:**
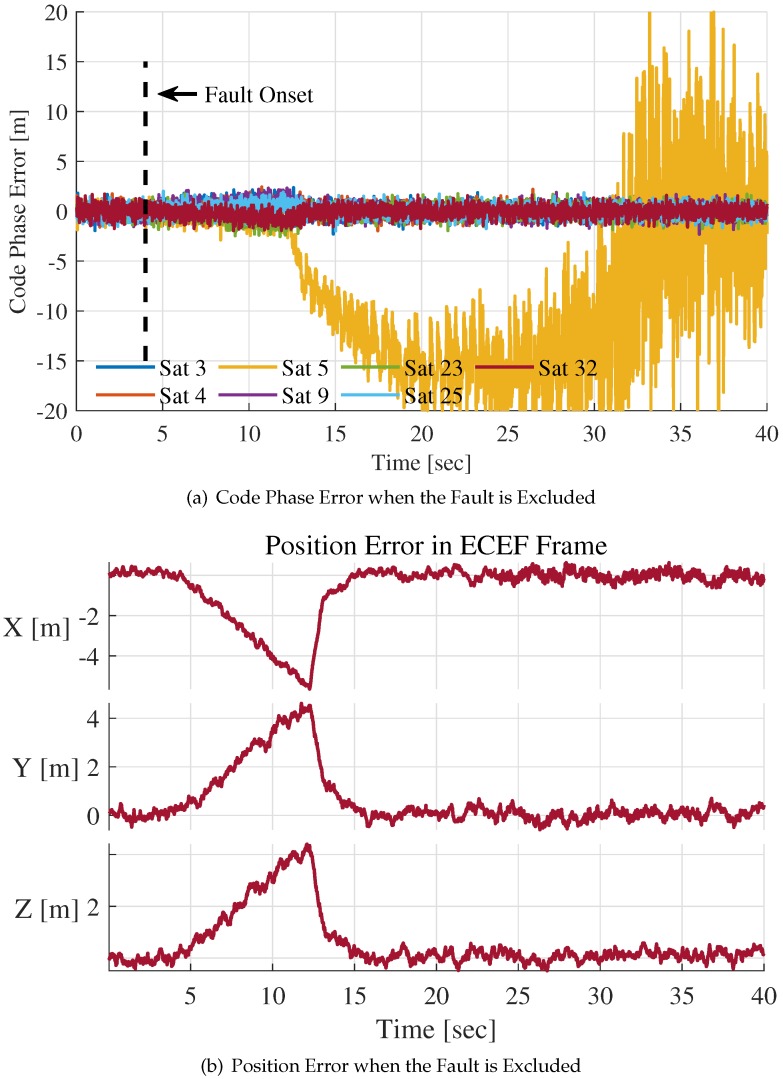
Code phase error estimations and position error for 1 m/s slowly growing error after fault exclusion.

In Figure 10 and Figure 11, the SGE fault is detected but not isolated from integration filter measurements. Figure 12a,b indicate the situation when the 1 m/s slowly growing error is isolated.

In Figure 12a, the SGE fault is detected at t=12.28 s. The absolute value of the faulty channel’s code phase error estimation increases with time, and the faulty channel loses lock gradually. Other channels are contaminated by the fault before the fault is detected, but they return to normal after t=12.28 s. The position errors shown in Figure 12b have similar changing trend. After the exclusion of the faulty channel, the mean value of the position errors comes back to zero by degrees, and the positioning result of the system becomes reasonable and correct. In conclusion, the fault detection and exclusion method is effective and efficient.

## 5. Conclusions

The deep integration system with a vector tracking architecture can promote tracking and positioning performance, especially in a degraded environment, but errors are easily propagated among channels in vector tracking architecture. Prior work on integrity research of the deep integration system is scant. A fault detection and exclusion method for step errors and SGEs is proposed in this paper. We utilize code phase error estimations and state corrections of the integration filter to form monitoring statistics. Simulations based on a software receiver platform were carried out for RAIM algorithm monitoring capability verification. Simulation results show that the FDE method proposed in this paper can detect and exclude the faults successfully and effectively. Since BDS has similar signal structure with GPS and Galileo satellite navigation system, the proposed method can also apply to these systems.

In future work, there are three aspects associated with the RAIM algorithm we need to explore. Firstly, the performance of the FDE method monitoring capability can be promoted by the rate detector algorithm. The algorithm may contribute to reduce detection time and improved sensitivity. Secondly, the RAIM algorithm can be influenced by unmodeled errors such as multipath and ionospheric scintillation. Extensive studies are required. The model of inertial sensor errors, a simple, monitoring ability test based on a specific MEMS IMU, will be carried out. Finally, the method proposed in this paper is only appropriate for a single fault scenario. FDE methods for the multiple faults scenario will be explored. 

## Figures and Tables

**Table 1:** State vector of the BDS/INS integration Kalman filter.

Symbol	Index	Description
δφ	1–3	Attitude error vector
$\delta v^{n}$	4–6	Velocity error vector
δp	7–9	Position error vector
$\varepsilon^{b}$	10–12	Gyroscope bias error vector
$\nabla^{b}$	13–15	Accelerometer bias error vector
$\delta b_{clk}$	16	Receiver clock bias error
$\delta d_{clk}$	17	Receiver clock drift error

**Table 2:** Accelerometer and gyroscope configuration parameters in simulation.

Parameters	Accelerometer	Gyroscope
Bias	4 mg	8 deg/h
Random walk noise	0.16 m/s/$\sqrt{h}$	0.16 deg/$\sqrt{h}$
Output rate	200 Hz	200 Hz

**Table 3:** Fault detection times of 30 Monte Carlo tests for SGEs.

Slope	Sample Mean	Sample Standard Deviation
1m/s	8.79s	0.76s
0.5m/s	17.74s	0.69s

## References

[1] Li Z., Chen W., Ruan R., Liu X. (2020). Evaluation of PPP-RTK based on BDS-3/BDS-2/GPS observations: a case study in Europe. GPS Solut..

[2] Gao Y., Liu S., Atia M.M., Noureldin A. (2015). INS/GPS/LiDAR integrated navigation system for urban and indoor environments using hybrid scan matching algorithm. Sensors.

[3] Gao G., Lachapelle G. INS-assisted high sensitivity GPS receivers for degraded signal navigation. Proceedings of the 19th International Technical Meeting of the Satellite Division of the Institute of Navigation.

[4] Kaplan E., Hegarty C. (2005). Understanding GPS: Principles and Applications.

[5] Groves P.D. (2013). Principles of GNSS, Inertial, and Multisensor Integrated Navigation Systems, Second Edition.

[6] Petovello M., Lachapelle G. (2006). Comparison of vector-based software receiver implementations with application to ultra-tight GPS/INS integration. Proc. ION GNSS.

[7] Edwards W.L., Clark B.J., Bevly D.M. Implementation details of a deeply integrated GPS/INS software receiver. Proceedings of the IEEE/ION Position, Location and Navigation Symposium.

[8] Xie F., Liu J., Li R., Jiang B., Qiao L. (2015). Performance analysis of a federated ultra-tight global positioning system/inertial navigation system integration algorithm in high dynamic environments. Proc. Inst. Mech. Eng. G J. Aerosp. Eng..

[9] Lashley M., Bevly D.M. (2008). A comparison of the performance of a non-coherent deeply integrated navigation algorithm and a tightly coupled navigation algorithm. Proc. Inst. Navig..

[10] Serant D., Kubrak D., Monnerat M., Artaud G., Ries L. Field test performance assessment of GNSS/INS ultra-tight coupling scheme targeted to mass-market applications. Proceedings of the 6th ESA Workshop on Satellite Navigation Technologies (Navitec 2012) & European Workshop on GNSS Signals and Signal Processing.

[11] Lashley M., Bevly D.M., Hung J.Y. Analysis of deeply integrated and tightly coupled architectures. Proceedings of the IEEE/ION Position, Location and Navigation Symposium.

[12] Ohlmeyer E.J. Analysis of an ultra-tightly coupled GPS/INS system in jamming. Proceedings of the IEEE/ION Position, Location, And Navigation Symposium.

[13] Parkinson B., Spilker J. (1996). Progress in Astronautics and Aeronautics: Global Positioning System: Theory and Applications.

[14] Bhattacharyya S., Gebre-Egziabher D. (2014). Integrity monitoring with vector GNSS receivers. IEEE Trans. Aerosp. Electron. Syst..

[15] Sturza M.A. (1988). Navigation system integrity monitoring using redundant measurements. Navigation.

[16] Parkinson B.W., Axelrad P. (1988). Autonomous GPS integrity monitoring using the pseudorange residual. Navigation.

[17] Bhatti U.I., Ochieng W.Y., Feng S. (2007). Integrity of an integrated GPS/INS system in the presence of slowly growing errors. Part I: A critical review. Gps Solut..

[18] Bhatti U.I., Ochieng W.Y., Feng S. (2007). Integrity of an integrated GPS/INS system in the presence of slowly growing errors. Part II: analysis. GPS Solut..

[19] Bhatti U.I., Ochieng W.Y., Feng S. (2012). Performance of rate detector algorithms for an integrated GPS/INS system in the presence of slowly growing error. GPS Solut..

[20] Walter T., Enge P. (1995). Weighted RAIM for precision approach. Proc. ION GPS.

[21] Pervan B.S., Lawrence D.G., Parkinson B.W. (1998). Autonomous fault detection and removal using GPS carrier phase. IEEE Trans. Aerosp. Electron. Syst..

[22] Hewitson S., Wang J. (2006). GNSS receiver autonomous integrity monitoring (RAIM) performance analysis. GPS Solut..

[23] Bhattacharyya S., Gebre-Egziabher D. (2010). Development and validation of parametric models for vector tracking loops. Navigation.

[24] Qin F., Zhan X., Zhang X. Detection and mitigation of errors on an ultra-tight integration system based on integrity monitoring method. Proceedings of the 26th International Technical Meeting of the Satellite Division of The Institute of Navigation.

[25] Zou X., Lian B., Wu P. (2019). Fault Identification Ability of a Robust Deeply Integrated GNSS/INS System Assisted by Convolutional Neural Networks. Sensors.

[26] Erickson J.W., Maybeck P.S., Raquet J.F. (2005). Multipath-adaptive GPS/INS receiver. IEEE Trans. Aerosp. Electron. Syst..

[27] Psiaki M.L. (2001). Smoother-based GPS signal tracking in a software receiver. Proc. ION GPS.

[28] Psiaki M.L. Extended Kalman filter methods for tracking weak GPS signals. https://www.researchgate.net/publication/266370295_Extended_Kalman_Filter_Methods_for_Tracking_Weak_GPS_Signals.

[29] Macchi F. (2010). Development and testing of an L1 combined GPS-Galileo software receiver. Ph.D. Thesis.

[30] Shin E.H. (2001). Accuarcy Improvement of Low Cost INS/GPS for Land Applications.

[31] Bhattacharyya S., Gebre-Egziabher D. (2014). Vector loop RAIM in nominal and GNSS-stressed environments. IEEE Trans. Aerosp. Electron. Syst..

[32] Angus J. (2006). RAIM with multiple faults. Navigation.

[33] Oh S.H., Hwang D.H. (2017). Low-cost and high performance ultra-tightly coupled GPS/INS integrated navigation method. Adv. Space Res..

